# Pharmacovigilance insights into drug-associated venous thromboembolism

**DOI:** 10.1097/JS9.0000000000002931

**Published:** 2025-07-02

**Authors:** Xiaojing Cai, Guoquan Chen, Haiping Wang, Ling Wang, Congli Hu

**Affiliations:** a Department of Pharmacy, The Third Affiliated Hospital of Wenzhou Medical University, Wenzhou, China; b Department of Pharmacy, Affiliated Jinhua Hospital, Medical College of Zhejiang University, Jinhua, China; c Department of Pharmacy, Longyan Second Hospital, Longyan, China; d School of Pharmaceutical Sciences, Zhejiang Chinese Medical University, Hangzhou, China; e Department of Pharmacy, Wenzhou TCM Hospital of Zhejiang Chinese Medical University, Wenzhou, China

**Keywords:** adverse events, FAERS, pharmacovigilance, venous thromboembolism

## Abstract

**Background:**

Venous thromboembolism (VTE), affecting 1–2 per 1000 adults annually, represents a major preventable cause of hospitalization and mortality. The use of specific medications is an acquired risk factor for VTE. This pharmacovigilance study systematically evaluated medication-associated VTE risk using the largest publicly available adverse event database.

**Methods:**

Disproportionality analysis of adverse event reports from the US Food and Drug Administration Adverse Event Reporting System was conducted between 2004 Q1 and 2024 Q3. Medications were stratified by Anatomical Therapeutic Chemical classification, with time-to-event analysis using Weibull distribution modeling (shape parameter β).

**Results:**

There were 168 960 reports associated with drug-associated VTE, encompassing 1718 medications. Of the 135 medications identified by disproportionality analysis as having a significant risk, 58 did not mention VTE in their package inserts. Antineoplastic and immunomodulating agents were found to have the largest number (64, 47.4%), followed by genito-urinary system and sex hormones (34, 25.2%), and blood and blood-forming organs (16, 11.9%). The shape parameter β of all cases was 0.649 (95% CI: 0.643–0.656), indicating an early failure pattern. The shortest drug-associated times were observed with andexanet alfa, recombinant FVIIa, and basiliximab. Females (55%) and the 45–64 age group (34%) were predominantly affected. Reports and deaths due to drug-associated VTE have increased over the years.

**Conclusion:**

A total of 135 medications showed positive signals for VTE (58 unmentioned in package inserts). The high-risk profile of anti-tumor agents and immunomodulators was highlighted. These findings provide robust data-driven guidance for clinical pharmacotherapy to mitigate VTE risks.

## Introduction

Venous thromboembolism (VTE), including pulmonary embolism and deep venous thrombosis, is the third most common cause of death worldwide^[1,2]^. The annual incidence of VTE varies by country, ranging from 1 to 2 per 1000 persons^[3]^. Although acquired risk factors such as surgery and malignancy^[4]^ are well-characterized, drug-associated VTE remains systematically underestimated^[5]^.

**HIGHLIGHTS**

- The present study identified 135 drugs with positive signals for venous thromboembolism (VTE), 58 of which did not mention in their package inserts.
- Anti-neoplastic and immunomodulators emerged as the dominant category, constituting 47.4% of VTE-associated drugs, with specific agents posing significant risks.
- Time-event analysis revealed risk patterns across drug categories, providing critical references for clinical drug use.
- These findings highlight the urgent need for regulatory actions, such as updating drug labels to reflect VTE risk profiles.

Several studies^[6–8]^ have indicated that certain medications, including hemostatic agents, oral contraceptives, and targeted cancer therapies, are associated with an increased risk of VTE. However, the absence of a comprehensive inventory of drug-associated VTE risks poses limitations to clinical risk stratification and prevention strategies, particularly for drugs with delayed thrombogenic effects.

Pharmacovigilance data^[9]^ are indispensable for post-market drug surveillance, capturing the nuances of real-world medication use more faithfully than laboratory or clinical trial results. This pharmacovigilance study aimed to offer comprehensive insights into the VTE risk of medications in real-world settings from the Food and Drug Administration (FDA) Adverse Event Reporting System (FAERS) database and identify medications with potential VTE risks that are not listed in the package insert. Reporting was conducted in accordance with the current best practices for transparent AI research, specifically adhering to TITAN guideline^[10]^.

## Materials and methods

### Data source

Data spanning from 2004 Q1 to 2024 Q3 were extracted from the FAERS database (https://fis.fda.gov/extensions/FPDQDE-FAERS/FPD-QDE-FAERS.html), covering demographics, drug information, adverse events, indications, report sources, and outcomes. Reports have been deduplicated following the FDA’s recommended method^[11]^. In particular, if CASEID was the same, the latest FDA_DT and highest PRIMARYID were chosen. The FAERS database was anonymized and exempted from the need for informed consent and institutional review board approval.

### VTE events and drug identification

Adverse events (AEs) in the FAERS database were classified using the Medical Dictionary for Regulatory Activities (MedDRA) version 27.0. VTE events were identified according to the preferred term (PT) level in the MedDRA terminology, and the code and PT names are shown in Supplemental Digital Content Table S1, available at: http://links.lww.com/JS9/E572.

Drugs with a reported role code as a “primary suspect” (PS) were included in this study. The Anatomical Therapeutic Chemical (ATC) classification system was used to code drugs and manually integrate synonyms with the same active ingredients to obtain the final list of causative drugs for VTE. For the same drug, all forms of drug names were gathered to increase accuracy, including drug code, brand name, active pharmaceutical ingredients, and generic name. We further reviewed the package inserts of these medications to ascertain the presence of VTE-related adverse drug events within the safety label.

### Disproportionality analysis

Disproportionality analysis^[12]^ was used to generate hypotheses on possible associations between drugs and VTE, including the ratio of reported odds (ROR)^[12]^, proportional reported ratio (PRR)^[13]^, Bayesian confidence propagation neural network (BCPNN)^[14]^, and multi-item gamma Poisson shrinker^[15]^. Multiple algorithms were combined to leverage their respective strengths, thereby enhancing the sensitivity and specificity of the signal detection. The calculation formula and criteria for positive signals are shown in Supplemental Digital Content Table S2, available at: http://links.lww.com/JS9/E572. A positive AE signal is generated only if the four algorithmic criteria are satisfied simultaneously. To further distinguish the potential VTE risk differences, drugs with potential VTE risk (positive signal) were divided into four levels (A, B, C, and D) according to the top 5%, 6%–20%, 21%–50%, and 51%–100% of ROR values, respectively^[16]^.

### Time-to-onset analysis

Time-to-onset (TTO) analysis was conducted for the positive signal drugs. TTO was calculated by determining the duration between the date of AE occurrence (EVENT_DT) and the initiation date of treatment (START_DT), according to the formula “START_DT- EVENT_DT + 0.5” as a general principle. Reports containing input errors, inaccurate date entries, or missing data were also excluded. The varying risk incidence increase or decrease in drug-associated VTE over time was determined and predicted using Weibull distribution^[17]^. The parametric distribution is characterized by the scale parameter α (representing the magnitude of ADRs occurring at the 63.2% quantile within the distribution function) and shape parameter *β*. The value of *β* < 1 suggested that the incidence of VTE decreased over time, indicating an early failure type.

### Statistical analysis

Descriptive analysis was performed to summarize the clinical features, including patient’ demographics, reporting country, outcomes, and occupation of reporters. Categorical variables were presented as frequencies and percentages, whereas continuous variables were described with medians and interquartile ranges. The research was performed according to the reporting of a disproportionality analysis for drug safety signal detection using individual case safety reports in pharmacovigilance^[18]^ (READUS-PV) (Supplemental Digital Content Table S3, available at: http://links.lww.com/JS9/E572). All analyses were performed using the R Software (version 4.3.1; R Foundation for Statistical Computing, Vienna, Austria).

## Results

### Clinical characteristics

**Figure 1.**
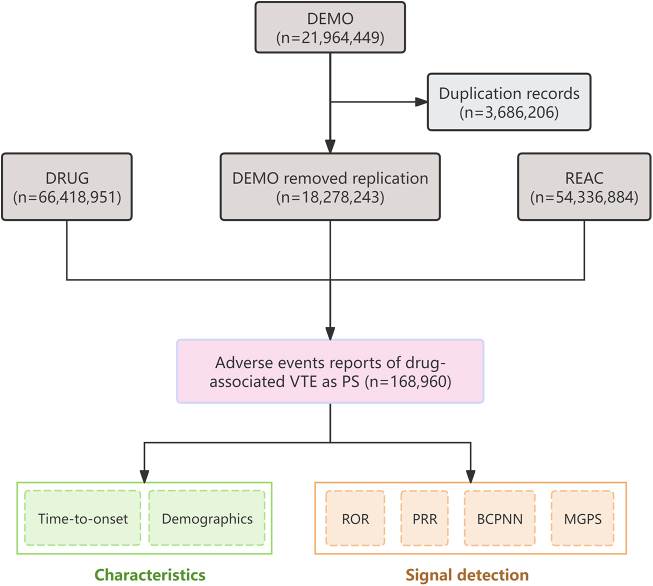
The flow diagram of the selection of drug-associated VTE reports.

VTE, venous thromboembolism; DEMO, demographic and administrative information; DRUG, drug details; RECT, adverse events; PS, primary suspect; ROR, the ratio of reported odds; PRR, proportional reported ratio; BCPNN, bayesian confidence propagation neural network; MGPS, multi-item gamma poisson shrinker.

**Figure 2.**
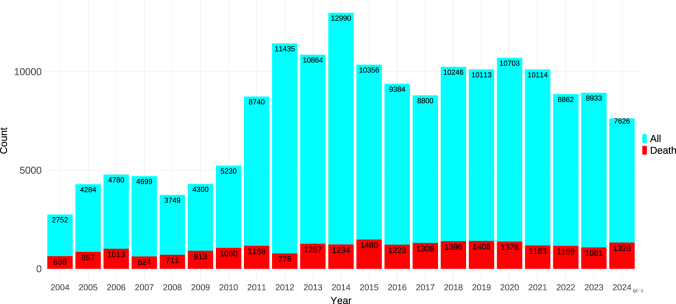
The annual distribution of drug-associated VTE reports and death reports from 2004 to 2024 Q3.

After removing deduplication, a total of 18 278 243 AE reports were included in the FAERS database from 2004 Q1 to 2024 Q3. There were 168 960 reports of drug-associated VTE, encompassing 1718 medications (Fig. 1). The demographic features of drug-associated VTE are described in Table 1. There was a higher proportion of reports on females (55% vs. 35%). In terms of patient age, the median age of the patients was 56 years, and patients with drug-associated VTE were mostly distributed in the 45–64 age groups (34%). Notably, most reports of drug-associated VTE were from the United States (57%), and exceeded those from France (6.1%), Germany (5.3%), Canada (5.2%), and the United Kingdom (5.1%). The occupation of the reporters was specified in 147 470 (87.3%) reports, of which 94 730 (64.2%) were submitted by health professionals. The most frequently reported outcomes were initial or prolonged hospitalization (45%), followed by other serious outcomes (32%), death (14%), and life-threatening outcomes (8.3%). As shown in Figure 2, from 2004 Q1 to 2024 Q3, the total number of cases increased significantly, reaching a maximum in 2014 (12 990 cases). Following the peak, the number of reports decreased and then stabilized. The number of deaths also increased over time, but at a much lower magnitude, maintaining a consistent proportion relative to the total.

VTE, venous thromboembolism.

**Table 1:** The demographic features of drug-associated VTE

Characteristic	Available number	Drug-associated VTE (*N* = 168 960)
Age (years), median (IQR)	119 751	56 (39, 69)
AGE_group, *n* (%)	119 751	
<18		4393 (3.7%)
≥75		17 449 (15%)
18–44		33 767 (28%)
45–64		40 734 (34%)
65–74		23 408 (20%)
Weight (kg), median (IQR)	34 209	80 (66, 105)
Sex, *n* (%)	168 960	
Female		93 385 (55%)
Male		59 467 (35%)
Unknown		16 108 (9.5%)
Top five report countries, *n* (%)	160 338	
United States		90 770 (57%)
France		9738 (6.1%)
Germany		8496 (5.3%)
Canada		8323 (5.2%)
United Kingdom		8234 (5.1%)
Outcomes, *n* (%)	166 614	
Hospitalization – initial or prolonged		74 623 (45%)
Other serious (important medical event)		53 213 (32%)
Death		23 205 (14%)
Life-threatening		13 882 (8.3%)
Disability		1340 (0.8%)
Required intervention to prevent		308 (0.2%)
Congenital anomaly		43 (<0.1%)
Occupation of reporters, *n* (%)	147 470	
Physician		56 437 (38%)
Consumer		42 486 (29%)
Other health-professional		27 601 (19%)
Pharmacist		10 692 (7.3%)
Lawyer		10 254 (7.0%)

IQR, interquartile range;VTE, venous thromboembolism.

### Disproportionality analysis

**Figure 3.**
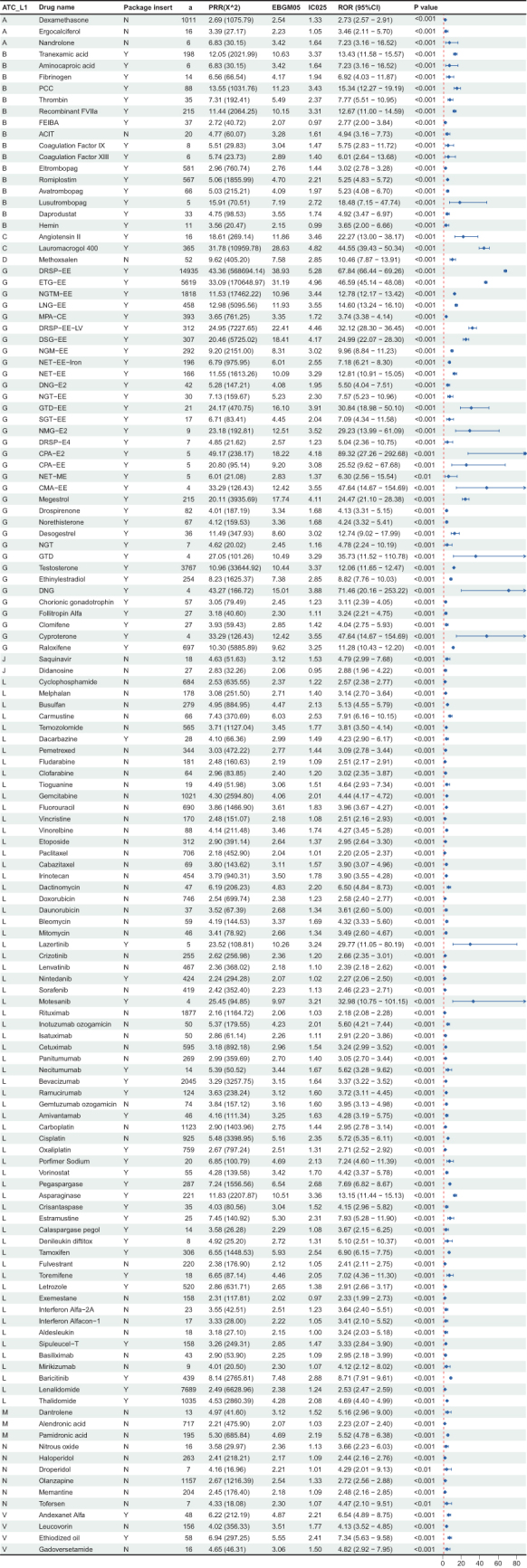
Forest plot and signal value of four disproportionality analysis methods for drugs causing drug-associated VTE. ATC, anatomical therapeutic chemical; L1, first level; VTE, venous thromboembolism; 95% CI, 95% confidence interval; FAERS, US Food and Drug Administration Adverse Event Reporting System; *X*^2^, chi-squared; PRR, proportional reported ratio; ROR, ratio of reported odds; IC expectations, information component; IC025, the lower limit of 95% CI of the IC; E(IC)EBGM, empirical Bayesian geometric mean; EBGM05, the lower limit of 95% CI of EBGM; A, alimentary tract and metabolism; B, blood and blood-forming organs; C, cardiovascular system; D, dermatologicals; G, genito urinary system and sex hormones; J, antiinfectives for systemic use; L, antineoplastic and immunomodulating agents; M, musculo-skeletal system; N, nervous system; V, various; FEIBA, factor VIII inhibitor bypassing activity; PCC, prothrombin complex concentrates; ACIT, Aprotinin; calcium chloride; Factor I (fibrinogen); thrombin; NMG, nomegestrol; E2, estradiol; GTD, gestodene; SGT, segesterone; EE, ethinyl estradiol; DNG, dienogest; MPA, medroxyprogesterone acetate; CE, conjugated estrogens; DRSP, drospirenone; LV, leucovorin; NGTM, norelgestromin; NGT, norgestrel; NET, norethindrone/norethisterone; ETG, etonogestrel; NGM, norgestimate; DSG, desogestrel; LNG, levonorgestrel; CMA, chlormadinone; CPA, cyproterone acetate.

**Figure 4.**
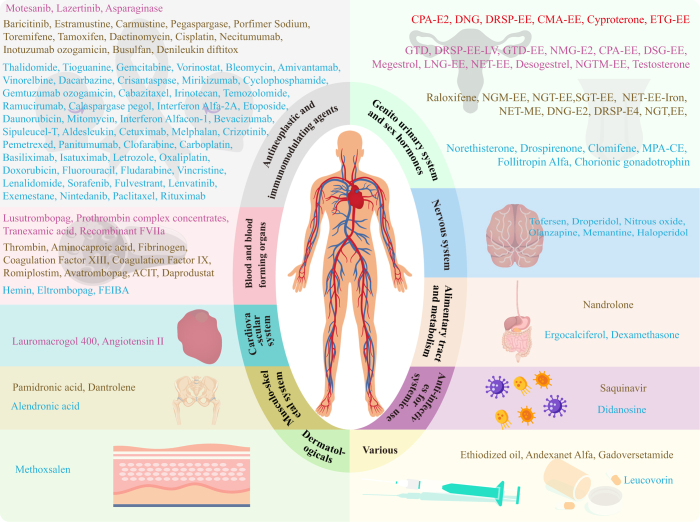
Classification of drugs causing drug-associated VTE according to the first level of ATC classification. Drugs with potential VTE are categorized into four levels: A, B, C, and D. These levels correspond to the top 5%, 6%–20%, 21%–50%, and 51%–100% of the ROR values, respectively. Level A is represented in red font, Level B in purple font, Level C in orange font, and Level D in blue font. FEIBA, factor VIII inhibitor bypassing activity; ACIT, aprotinin; calcium chloride; Factor I (fibrinogen); Thrombin; NMG, nomegestrol; E2, estradiol; GTD, gestodene; SGT, segesterone; EE, ethinyl estradiol; DNG, dienogest; MPA, medroxyprogesterone acetate; CE, conjugated estrogens; DRSP, drospirenone; LV, Leucovorin; NGTM, norelgestromin; NGT, norgestrel; NET, norethindrone/norethisterone; ETG, etonogestrel; NGM, norgestimate; DSG, desogestrel; LNG, levonorgestrel; CMA, chlormadinone; CPA, cyproterone acetate.

Disproportionality analyses were conducted for medications with reported cases, identifying 135 medications with positive signals (Fig. 3). Medications were classified into ten distinct categories according to the first level of the ATC classification system. Specifically, antineoplastic and immunomodulating agents (L) were found to have the largest number (64 drugs, 47.4%), followed by genito-urinary system and sex hormones (G) (34 drugs, 25.2%), blood and blood-forming organs (B) (16 drugs, 11.9%), and nervous system (N) (6 drugs, 4.4%). The other groups included various (V), alimentary tract and metabolism (A), musculo-skeletal system (M), cardiovascular system (C), anti-infectives for systemic use (J), and dermatologicals (D) (Fig. 4). The specific classification and summaries of the aforementioned medications related to drug-associated VTE were detailed in Supplemental Digital Content Table S4, available at: http://links.lww.com/JS9/E572.

The 64 antineoplastic and immunomodulating agents were further divided into ten subgroups at the third level of the ATC classification, including other antineoplastic agents (L01X, 11drugs, 17.2%), monoclonal antibodies and antibody drug conjugates (L01F, 10 drugs, 15.6%), alkylating agents (L01A, 6 drugs, 9.4%), antimetabolites (L01B, 6 drugs, 9.4%), plant alkaloids and other natural products (L01C, 6 drugs, 9.4%), protein kinase inhibitors (L01E, 6 drugs, 9.4%), cytotoxic antibiotics and related substances (L01D, 5 drugs, 7.8%), hormone antagonists and related agents (L02B, 5 drugs, 7.8%), immunosuppressants (L04A, 5 drugs, 7.8%), and immunostimulants (L03A, 4 drugs, 6.3%). Genito-urinary system and sex hormones involving 34 medications could be further divided into seven subgroups, including hormonal contraceptives for systemic use (G03A, 26 drugs, 76.5%), gonadotropins and other ovulation stimulants (G03G, 3 drugs, 8.8%), androgens (G03B, 1 drug, 2.9%), estrogens (G03C, 1 drug, 2.9%), progestogens (G03D, 1 drug, 2.9%), antiandrogens (G03H, 1 drug, 2.9%), and other sex hormones and modulators of the genital system (G03X, 1 drug, 2.9%).

The results of VTE risk stratification are detailed in Figure 4, in which levels A, B, C, and D contained 6, 21, 40, and 68 drugs, respectively. The top three drugs with the highest risk were cyproterone acetate and estradiol combination (CPA-E2) (IC: 5.62; ROR: 89.32; *P*: < 0.001), dienogest (DNG) (IC: 5.44; ROR: 71.46; *P*: < 0.001), and drospirenone and ethinylestradiol combination (DRSP-EE) (IC: 5.31; ROR: 67.84; *P*: < 0.001).

### TTO analysis

**Figure 5.**
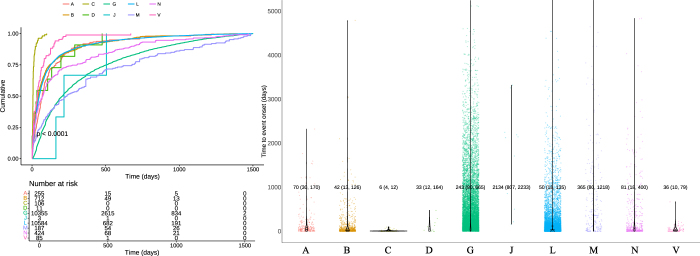
Cumulative risk curve and violin plots comparing drug-associated VTE onset times for drugs classified by the first level of ATC classification. (Left) Cumulative risk curve for drug-associated VTE onset times for drugs classified by the first ATC classification level. (Right) Comparison of drug-associated VTE onset times for drugs classified according to the first ATC classification level. A, alimentary tract and metabolism; B, blood and blood-forming organs; C, cardiovascular system; D, dermatologicals; G, genitourinary system and sex hormones; J, antiinfectives for systemic use; L, antineoplastic and immunomodulating agents; M, musculoskeletal system; N, nervous system; V, various.

There were 23 963 cases of drug-associated VTE documenting onset time in the FAERS database, with a median onset time of 105 days (IQR 34–365). Among the medications used in more than 10 reported cases, andexanet alfa exhibited the shortest median time-to-onset of 4 days (IQR: 1–12), followed by recombinant FVIIa (median 5 days, IQR: 3–14), and basiliximab (median 5 days, IQR: 4–27). Additionally, based on the classification of the first level of ATC, the cumulative risk curve results revealed significant differences in drug-associated onset time among different categories of drugs (*P* < 0.0001) (Fig. 5A). The shortest TTO was observed in the cardiovascular system group (median 6 days, IQR: 4–12), the median TTO was 50 and 243 days for the antineoplastic and immunomodulating agents (IQR: 18–135 days) and genito-urinary system and sex hormones (IQR: 90–665 days), respectively (Fig. 5B). The TTO profile was exhaustively evaluated through integration of the median, quartile, and Weibull shape parameter tests. The shape parameter β of all cases was 0.649 (95% CI: 0.643–0.656). This suggests that the onset of VTE associated with these medications followed an early failure mode. For the 55 medications, the 95% CI of the *β* value encompassed 1, signifying a random failure pattern. The *β* value of dacarbazine was 1.963 (95% CI: 1.220–2.707), indicating a wear-out failure pattern. The median and Weibull parameters of the individual positive medications are listed in Supplemental Digital Content Table S5, available at: http://links.lww.com/JS9/E572.

## Discussion

To the best of our knowledge, this study is the most extensive and methodical pharmacovigilance examination to assess the risk of drug-associated VTE from the FAERS database, providing valuable real-world data validation for the revision and supplementation of package inserts of post-marketed medications. A total of 135 medications showed positive signals for VTE, 58 of which did not mention VTE in their package inserts.

Thrombosis is the second leading cause of death in patients with known cancer^[19]^, with risks varying according to tumor site and stage. Patients with hematologic malignancies had the highest risk, followed by those with lung and gastrointestinal cancer^[20]^. Age, extent and site of cancer, and antineoplastic medications play synergistic roles in thrombosis development^[21]^. Current categories of these medications include cytotoxic agents, hormonal therapies, targeted therapies, and immunotherapies. There are 24 cytotoxic medications with VTE-positive signals lacking warnings in their package inserts, including five alkylating agents (cyclophosphamide, temozolomide, busulfan, melphalan, and carmustine), six plant alkaloids (paclitaxel, irinotecan, etoposide, vincristine, vinorelbine, cabazitaxel), six antimetabolites (gemcitabine, fluorouracil, pemetrexed, fludarabine, clofarabine, and thioguanine), five cytotoxic antibiotics (doxorubicin, bleomycin, actinomycin D, mitomycin, and daunorubicin), and two platin-based agents (carboplatin, and cisplatin).

Among patients with non-hematologic malignancies, a cohort study^[22]^ identified irinotecan therapy as an independent risk factor for VTE development (hazard ratio [HR], 1.89; 95% CI: 1.29–3.59). Patients with advanced pancreatic cancer^[23]^ treated with first-line palliative FOLFIRINOX (oxaliplatin, irinotecan, leucovorin, and fluorouracil) or gemcitabine/nab-paclitaxel exhibited a high risk of VTE. Transplant-eligible multiple myeloma patients randomly assigned to cyclophosphamide, vincristine, doxorubicin, and dexamethasone induction had a higher risk of VTE than patients treated with cyclophosphamide, thalidomide, and dexamethasone (aHR: 1.46; 95% CI: 1.11–1.93). A meta-analysis^[24]^ including 4845 patients from 19 trials showed that the use of gemcitabine tends to increase the risk of VTE, compared with non-gemcitabine-based therapy. There are scant controlled clinical data on the effect of fluorouracil on VTE risk, which is further complicated by its frequent use as part of combination chemotherapy regimens.

A Phase III prospective trial^[25]^ revealed that the regimen including doxorubicin was linked to a significantly elevated VTE risk compared to the regimen without doxorubicin in multiple myeloma patients (16.0% vs. 2.5%). A meta-analysis showed a significantly increased risk of VTE in patients treated with cisplatin-based chemotherapy compared with non-cisplatin-based regimens^[26]^. The PROTECHT^[27]^ score, which expands the Khorana score to include chemotherapy factors, lacks adequate validation. The underlying prothrombotic mechanisms are poorly understood; they appear to induce endothelial injury^[28]^, reduce the protein C pathway, increase tissue factor procoagulant activity, and activate platelets.

Hormonal therapy^[29]^ is an effective and non-toxic therapy that improves the clinical outcomes of certain hormone-dependent cancers. Our study revealed that tamoxifen, letrozole, fulvestrant, exemestane, and toremifene were associated with an increased risk of VTE, with the risk not mentioned in the package inserts for fulvestrant and exemestane. Clinicians need to monitor the risk of VTE in breast cancer patients receiving hormonal therapy, and the risk appears to be greatest in the short term immediately following initiation^[30]^. A prospective cohort study^[31]^ demonstrated that initiating tamoxifen therapy is associated with increased thrombin generation and enhanced protein C resistance.

Previous studies^[32–34]^ shed some light on the interaction between inflammation and thrombosis, revealing the pro-thrombotic activity of some cytokines (including IFNγ, IL-6, CCL2, IL-17A, IL-9, IL-1β, and TGF-β). Our research uncovered a potential association between three immunomodulators (interferon alfa-2A, aldesleukin, and interferon Alfacon-1) and two immunosuppressants (basiliximab and mirikizumab) with increased VTE risk, with mechanisms that require additional exploration. The VTE risk associated with baricitinib^[35,36]^, thalidomide^[37,38]^, and lenalidomide^[39,40]^ has been well established. Patients treated with thalidomide showed increased platelet P-selectin exposure, endothelial damage, and increased abundance of platelet-monocyte aggregates^[38,41]^. Sipuleucel-T^[42,43]^ is the only approved therapeutic vaccine for metastatic castration-resistant prostate cancer. Whether sipuleucel-T induces a generalized inflammatory response associated with thromboembolic events has not been reported.

The development of targeted therapies has led to a paradigm shift in anti-neoplastic treatment, moving toward more personalized cancer therapies^[44]^. This study identified the prothrombotic effects of six monoclonal antibodies (rituximab, inotuzumab ozogamicin, isatuximab, cetuximab, gemtuzumab ozogamicin, and Panitumumab) and four tyrosine kinase inhibitors (lenvatinib, sorafenib, motesanib, and crizotinib). Pooled data^[45]^ from 17 RCTs, including 12 870 patients, indicated that a higher rate of severe VTE was observed with cetuximab compared to the respective control (RR: 1.46; 95% CI: 1.26–1.69). Further research is needed to elucidate the potential causes of elevated VTE risk, which may include the direct effects of targeted therapies, molecular changes and mutations in tumors, or prolonged patient survival.

Combined oral contraceptives are well-researched risk factors for VTE^[7]^. Research has revealed that combined oral contraceptives, gonadotropins, androgens, antiandrogens, progestogens, and estrogens are associated with an increased risk of VTE. Controlled ovarian hyperstimulation, an integral component of assisted reproductive technology, is linked to a higher VTE risk, possibly by causing elevated platelet counts, decreased antithrombin, and increased coagulation factor levels^[46]^. Some studies^[47,48]^ suggested that the progestin component may affect VTE risk along with estrogen, although the data were not uniformly consistent. EE-LNG and EE-NGM have been perceived as the safer choice for first-line contraception. A higher VTE risk for EE-LNG than for EE-NGM was identified in the present study. The increased risk of VTE associated with testosterone replacement treatment may be a consequence of polycythemia. However, a recent meta-analysis^[49]^ revealed that there was no significant association between testosterone and VTE in men with pre-treatment total testosterone levels <12 nmol/L. It’s important to note that the VTE risk associated with sex hormone use is also affected by factors such as age, smoking, obesity, and VTE history.

Sixteen specific antihemorrhagic medications were associated with an elevated risk of VTE. Thrombotic complications of antihemorrhagic medications are reasonable concerns, and may occur due to drug-associated thrombocytosis^[50]^, hypercoagulability^[51]^, and fibrinolytic inhibition^[6,52]^. It is critical to adhere to the indications, dosage, and duration recommendations for antihemorrhagics to minimize the risk of VTE. A significant correlation was identified between three atypical antipsychotic medications (olanzapine, haloperidol, and droperidol) and VTE, consistent with previous research^[53]^. Potential mechanisms may involve antipsychotic-induced adverse effects, such as sedation and metabolic alterations leading to weight gain, which collectively promote venous stasis through reduced mobility^[54]^. In addition, psychiatric disorders could increase the risk of VTE, particularly during the acute phase when patients may require physical restraint due to uncontrollable behavior^[55]^.

Other medications that may increase the risk of VTE include anti-osteoporosis drugs, sclerosing agents, contrast agents, glucocorticoids, and antiretroviral medications. A meta-analysis^[56]^ of 28 RCTs revealed that VTE rates may be increased in the polidocanol group (RR 5.10, 95% CI 1.30–20.01). VTE associated with ethiodized oil^[57]^ may result from inadvertent intravascular injection or intravasation, pending further confirmation of causality. Dexamethasone is an independent predictor^[58]^ of VTE in patients with multiple myeloma, with a dose-dependent pattern.

This study has several limitations that should be acknowledged. First, the voluntary nature of spontaneous reporting databases poses inherent challenges in terms of inaccuracies, incompleteness, and possible under-reporting, especially for drugs that have been marketed for a long time. Nonetheless, this substantial caseload provides valuable insights into drug-associated VTE. Second, VTE is associated with Virchow’s triad^[55]^ (hypercoagulability, blood flow stagnation, and venous wall damage). However, the FAERS database lacks essential variables for comprehensive VTE risk assessment, precluding the complete elimination of unmeasured confounding factors. Finally, as a passive surveillance system confined to adverse event case submissions, it is not feasible to calculate the incidence of drug-associated VTE. The disproportionality analysis demonstrated a correlation, but did not confirm causation. Further validation is necessary to enhance the robustness and reliability of the findings.

## Conclusion

This study reviewed and analyzed VTE reports using data from the FAERS database over the period from 2004 Q1 to 2024 Q3. Overall, 135 medications showed positive signals for VTE, 58 of which did not mention VTE in their package inserts. The high-risk profile of anti-tumor agents and immunomodulators, along with risk patterns across different drug categories, was identified. Additionally, the underlying mechanisms of drug-associated VTE were systematically investigated. These findings provided strong evidence for drug safety monitoring and package insert updates.

## Data Availability

The datasets generated and/or analyzed during the current study are available in the US FAERS database (https://fis.fda.gov/extensions/FPD-QDE-FAERS/FPD-QDE-FAERS.html). The code generated and/or analyzed in the current study is available from the corresponding author on reasonable request.
